# Decoding Anti–Substance Use Public Service Announcements: Content Analysis Grounded in the Elaboration Likelihood Model and Extended Parallel Process Model

**DOI:** 10.2196/85703

**Published:** 2026-05-14

**Authors:** Jiawei Liu, Zhaomeng Niu

**Affiliations:** 1 Department of Communication Studies, Ernestine M. Raclin School of the Arts, Indiana University South Bend, South Bend, IN, 46615, United States; 2 Health Informatics, School of Health Professions, Rutgers University, New Jersey, NJ, 08901, United States

**Keywords:** extended parallel process model, elaboration likelihood model, public service announcement, substance use, content analysis

## Abstract

**Background:**

Tobacco, alcohol, and illicit drug use continue to pose substantial public health challenges in China. Although public service announcements (PSAs) are widely used for prevention, little is known about how these messages are constructed or the extent to which they draw on established health communication theories.

**Objective:**

This exploratory study aimed to characterize the design features of anti–substance use PSAs in China, assess their use of constructs from the extended parallel process model (EPPM) and the elaboration likelihood model (ELM), and compare patterns across anti–substance use PSAs.

**Methods:**

We conducted a content analysis of 89 publicly available anti–substance use PSAs produced in mainland China. Messages were identified via major Chinese video platforms and institutional websites and then screened using predefined eligibility criteria. Variables captured message source, intended audience, framing, substance depiction, cultural appeals, and EPPM and ELM components. Frequencies and proportions were calculated, and *χ*^2^ tests were used to examine differences by PSA type. To account for multiple comparisons, *P* values were adjusted using the Holm-Bonferroni correction.

**Results:**

Most PSAs did not identify a target audience (54/89, 60.7%), and public security departments were the most common sponsors (n=37, 41.2%), while none were sponsored by public health agencies. Theory use was selective: response efficacy (n=63, 70.8%) and perceived severity (n=55, 61.8%) appeared more often than self-efficacy (n=45, 50.6%) and perceived susceptibility (n=34, 38.2%); peripheral cues (n=79, 88.8%) were more common than central route cues (n=16, 18%). Differences across PSA types were observed in sponsorship, message features, and theoretical constructs. After adjustment for multiple comparisons, associations involving sponsoring organizations (public security departments and Chinese media) and perceived susceptibility remained statistically significant (all adjusted *P*=.01). Antidrug PSAs were predominantly associated with public security sponsorship, whereas antialcohol and antitobacco PSAs were more frequently linked to Chinese media sources. Perceived susceptibility cues were more common in antismoking PSAs than in antidrug PSAs, while other differences in framing, substance cues, cultural appeals, and ELM or EPPM constructs were not statistically significant after adjustment.

**Conclusions:**

Anti–substance use PSAs in China were characterized by limited audience segmentation and uneven use of theory-based persuasive strategies. Observed differences across alcohol-, tobacco-, and drug-focused messages suggest that PSA design may be shaped not only by partial application of communication theory but also by institutional influences and substance-specific contexts. These findings highlight the need for more context-sensitive and theory-informed approaches to anti–substance use PSA design in China.

## Introduction

Substance use, including tobacco use, alcohol use, and illicit drug use, remains a major public health concern worldwide and in China. According to the World Health Organization [1,2], in 2019 alone, alcohol accounted for 2.6 million deaths (4.7% of global deaths), while drug use led to 600,000 deaths, and tobacco killed around 8 million people worldwide, including from secondhand smoke. In China, 26.6% of adults smoke, including 50.5% of men and 2.1% women [3]. While global alcohol consumption declines, China’s per capita intake is rising, with alcohol-related illnesses causing 6% of male and 1% of female deaths [4]. Although drug use is comparatively low, 896,000 users were reported by the end of 2023 [5]. Beyond their direct health consequences, these forms of substance use impose broader costs on families, communities, and health systems.

Mass media campaigns have long been used to raise awareness of substance-related harms and promote prevention and cessation behaviors [6-8]. Among these communication strategies, public service announcements (PSAs) are especially important because they can reach broad audiences through repeated exposure and accessible message formats. In China, anti–substance use PSAs are typically broadcast through various media channels, including television, subway and bus advertisements, and online platforms [9,10]. However, limited research has examined how these PSAs are designed and what types of persuasive strategies they adopt. Addressing this gap is essential, as PSA effectiveness depends not only on exposure but also on the way messages are crafted, framed, and directed.

The importance of integrating theory into health campaign messaging is well documented and foundational to this study. In particular, elaboration likelihood model (ELM) [11] and the extended parallel process model (EPPM) [12,13] provide useful frameworks for examining the persuasive design of health messages, including those related to cancer prevention, vaccination, and healthy eating [14-18]. The ELM explains attitude change through dual routes of persuasion: the central route, which involves careful scrutiny of message arguments leading to durable attitude shifts, and the peripheral route, which relies on superficial cues such as endorsements or slogans. The ELM has been widely applied to understand how individuals process health information and form attitudes toward a range of health-related behaviors [16-19]. Alongside the ELM, the EPPM focuses on how people respond to fear-based messages by evaluating the threat (perceived severity and susceptibility) and their ability to act (self-efficacy and response efficacy), which together affect their motivation and capacity to adopt recommended health behaviors [20,21]. Prior studies have shown that these theoretical constructs are valuable for analyzing how health messages communicate risks and motivate behavior change [16,19-25]. In the context of anti–substance use PSAs, these frameworks are also relevant because such messages often rely on fear appeals, efficacy messages, spokespersons, slogans, and other persuasive cues to influence public attitudes and behaviors.

In addition to identifying theory-based persuasive appeals, it is also important to examine broader message design features that may shape how such strategies are embedded and interpreted in PSAs. For example, these include the message source, the intended audience, the way the message is presented, and the cultural values it conveys. Specifically, the sponsoring organization may influence how credible or authoritative a PSA appears [26,27], whereas target audience specification reflects the degree of audience segmentation in message design [28]. Framing strategy and the depiction of substance use may further shape how risks and recommended behaviors are presented, including whether a message emphasizes harms, benefits, or potentially counterproductive cues [29,30]. Cultural appeals are also particularly relevant in the Chinese context, where public health communication has often emphasized collectivist values, prioritizing social responsibility and collective well-being, as reflected in prior public health practices such as responses to the COVID-19 pandemic [31]. Such a context may shape how anti–substance use PSAs are designed. This study examined whether such messages also emphasized collective well-being or individual-level concerns. Examining these features alongside ELM and EPPM constructs can therefore provide a more complete understanding of how anti–substance use PSAs in China are designed and how they may be improved in practice.

Overall, this study examined both the message design features and the theory-based persuasive strategies embedded in Chinese anti–substance use PSAs. Specifically, we analyzed message sources, intended audience, substance type, framing strategy, substance use depiction, and cultural appeals as message design features (Research Question 1). We then assessed the extent to which the PSAs incorporated threat and efficacy components drawn from the EPPM (Research Question 2) and central and peripheral route cues based on the ELM (Research Question 3). In addition, we examined how these message design features and theoretical constructs varied across antialcohol, antitobacco, and antidrug PSAs (Research Question 4). This study combined descriptive and theory-driven analyses to provide a more practically useful account of how anti–substance use PSAs in China are designed and where future campaign development may be strengthened.

## Methods

This study conducted a content analysis to examine the characteristics of anti–substance use PSAs (targeting alcohol, tobacco, or drugs) and their alignment with the key constructs of the EPPM and ELM. The sampled PSAs were created in Chinese by organizations based in mainland China and were disseminated to the general public.

### Search Strategy

As no national or regional database of PSAs in China was available during the study, anti–substance use PSAs produced in mainland China were identified by systematic searches of multiple online platforms and institutional websites. Searches were conducted across major Chinese video-sharing platforms, including BiliBili, iQIYI, Youku, and Tencent Video. These platforms were selected because they are among the most widely used video-hosting services in China and are commonly used by governmental and public health organizations to disseminate video-based campaign materials. In addition, official websites of governmental and public health institutions, including national and local antidrug offices and Centers for Disease Control and Prevention, were also searched to improve coverage.

Searches were performed using combinations of Chinese keywords related to substance use and public service messaging. These include terms related to alcohol use (ie, 饮酒，喝酒，酒，酒精), “tobacco” (ie, 吸烟，抽烟，烟，烟草), and “drug” (ie, 毒品，禁毒), combined with the term “public service announcement” (ie, 公益广告，公益宣传片). Keywords were entered in combination using platform search functions (eg, “禁毒 公益广告”). For each keyword combination, results returned by the platform’s default search interface were screened. Refer to Table S1 in Multimedia Appendix 1 for search strategy details.

The search process was conducted in multiple rounds between December 2023 and April 2024. An initial set of platforms (ie, major Chinese video-sharing platforms) and keywords were used, and additional sources (eg, governmental and public health websites) were incorporated when initial searches yielded a limited number of eligible PSAs. These adjustments were guided by predefined inclusion criteria and team discussions to improve coverage while maintaining consistency.

PSAs were included regardless of their production or publication date, as anti–substance use PSAs in mainland China were not systematically archived in a centralized database, and key information such as production dates and campaign affiliations is often not consistently reported across sources. Therefore, all eligible PSAs available up to April 2024 were included. Duplicate videos were identified across platforms and were removed based on title, visual content, and source information prior to analysis.

### Sample and Screening

**Figure 1.**
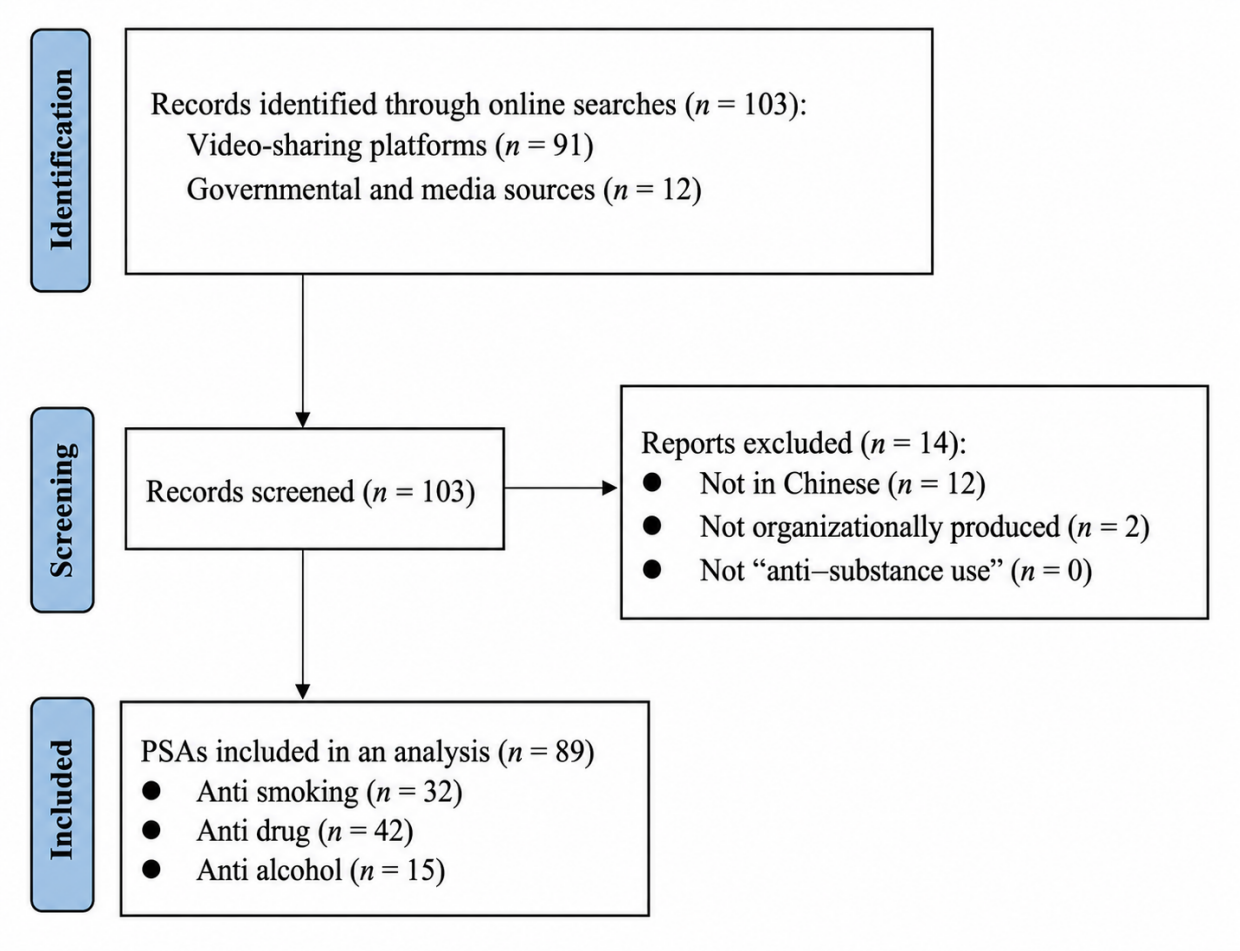
Flow diagram of the identification, screening, and inclusion of anti–substance use public service announcements (PSAs) produced in mainland China, based on multiplatform online searches conducted between December 2023 and April 2024.

A total of 103 anti–substance use PSAs were initially identified. All identified PSAs were screened according to specific criteria: (1) they had to be in Chinese, (2) they had to be produced by an organizational entity in mainland China (excluding school projects or personal creations), and (3) they had to focus on anti–substance use (ie, alcohol, tobacco, or drugs). The screening process was conducted by 2 trained graduate student researchers using a shared protocol. Each PSA was independently reviewed for eligibility, and discrepancies were resolved through research team discussion. Final inclusion decisions were reviewed and confirmed by the research team. After screening, 89 PSAs met the inclusion criteria and were retained for analysis. A flow diagram of the study selection process is presented in Figure 1.

### Measures

This study included 11 variables, all of which were categorical. To examine the characteristics of the sampled anti–substance use PSAs, we coded for the sponsoring organization, target audience, presence of substance cues, substance addiction type, framing strategy, and cultural value. For the *sponsoring organization*, PSAs were categorized into 6 types, including Chinese media, Chinese government, public welfare organizations, public health agencies, commercial enterprises, and those without a clearly identified sponsoring organization. Regarding the *target audience*, PSAs were coded into 4 groups: minors, young and middle-aged individuals, older adults, and those without a clearly defined target audience. For the presence of *substance cues*, PSAs were categorized based on whether they depicted specific substance addiction behaviors such as smoking, drinking, or drug use. This resulted in 2 categories: PSAs with substance cues and those without substance cues. *The type of substance addiction addressed* was classified as either focusing on a single substance use or polysubstance use. The *framing strategy* was classified as either positive framing or negative framing. In terms of *cultural values*, the PSAs were coded to reflect either a collectivist cultural perspective or an individualistic cultural perspective.

In addition, we examined how the sampled PSAs aligned with the ELM and EPPM. By analyzing the key constructs of the ELM, PSAs were coded based on their use of the *central route cues*, which feature clear and detailed arguments, or the *peripheral route cues*, which rely on credible sources or appealing presentation styles. By examining the key constructs of the EPPM, the PSAs were analyzed for perceived threat cues, including *perceived severity cues* and *perceived susceptibility cues,* and perceived efficacy cues, which included *response efficacy cues* and *self-efficacy cues*. Refer to Table 1 for more details.

**Table 1.** Coding categories, operational definitions, and intercoder reliability for variables used in a content analysis of anti–substance use public service announcements (PSAs) from mainland China (N=89).

Variables and measured concept		Operational definition	Cohen κ
Sponsoring organization			
Chinese media		PSAs produced and disseminated by media organizations based in China, including television networks, radio stations, and online news platforms	0.971
Public security departments		PSAs developed by public security departments responsible for addressing substance-related crimes and for substance use prevention and education	1.000
Chinese government		PSAs produced by various government departments or agencies at the national or local level, excluding public security departments	1.000
Public health agency		PSAs developed by organizations dedicated to public health, including health departments and nonprofit organizations focused on health promotion and disease prevention	0.000
Public welfare organization		PSAs produced by nongovernmental organizations and charities that focus on social welfare, community support, and public service initiatives	1.000
Commercial enterprises		PSAs created by businesses or corporations for promotional purposes	1.000
No sponsoring organization		No clear sponsoring organization or entity is identified	1.000
Target audience			
Minor		Visuals or audio indicate that the target audience is individuals aged <18 years	1.000
Young and middle-aged individuals		Visuals or audio indicate that the target audience is individuals aged between 18 and 64 years	0.979
Older adults		Visuals or audio indicate that the target audience is individuals aged ≥65 years	1.000
No clearly defined target audience		The PSA does not specify or clearly define a particular age group	1.000
The presence of substance cue			
Presence		Message depicts the use of alcohol, tobacco, or drugs	1.000
Absence		No depiction of alcohol, tobacco, or drug use	1.000
Addiction intervention scope			
Single substance addiction		The message presents a single type of substance addiction, such as smoking, drinking, or drug use	1.000
Polysubstance addiction		The message presents multiple types of substance addictions, including smoking, drinking, and drug use	1.000
Framing strategy			
Positive framed		Messages that highlight the benefits or positive outcomes of avoiding substance use	1.000
Negative framed		Messages that focus on the negative consequences or risks associated with substance use	1.000
Cultural value			
Collectivist		Messages discuss the benefits of avoiding substance use or the harms for family, community, or society	1.000
Individualistic		Messages discuss the personal benefits of avoiding substance use or the harms associated with it	1.000
EPPM^a^ constructs			
Threat appraisal			
Perceived severity		Audio or visuals illustrate the seriousness of the consequences of substance use	1.000
Perceived susceptibility		Messages discuss the likelihood of substance use	1.000
Efficacy appraisal			
Response efficacy		Confirmation that the proposed action will help avoid the harm	1.000
Self-efficacy		Advice on actions people can take to prevent substance use or abuse	1.000
ELM^b^ constructs			
Central route cue		Use of factual evidence, detailed explanations, counterarguments, and relevant statistics	0.971
Peripheral route cue		Use of attractive spokespeople, emotional appeals (such as fear and guilt), and celebrity endorsements	0.962

a EPPM: extended parallel process model.

b ELM: elaboration likelihood model.

### Coding Procedure

Two trained graduate students coded the data. The coders completed 3 separate rounds of coding: 10 videos in the first round, another 10 in the second, and an additional 15 in the third, representing 39.3% (34.97/89) of the total sample of PSAs. Coding results were compared and discussed, and intercoder reliability was assessed using Cohen κ. The Cohen κ values ranged from 0.928 to 1, indicating an acceptable level of reliability [32]. Further details are provided in Table 1. After satisfactory intercoder reliability was established, the remaining PSAs were allocated between the 2 coders and independently coded using the finalized coding scheme.

### Statistical Analyses

To address the research questions, we conducted both descriptive and inferential statistical analyses to examine the distribution of message design features and theory-based constructs in anti–substance use PSAs, as well as their variation across different PSA types. For Research Questions 1 to 3, descriptive statistics were used to summarize the presence of key message features and theoretical constructs, including sponsoring organization, target audience, framing strategy, substance depiction, cultural appeals, and ELM and EPPM components. For Research Question 4, *χ*² tests of independence were performed to examine whether these message features and theoretical constructs differed across PSA types (antialcohol, antitobacco, and antidrug). This approach was appropriate, given that all variables were categorical. For each significant association, Cramer V was calculated to assess effect size. To account for the potential inflation of type I error due to multiple comparisons, *P* values were adjusted using the Holm-Bonferroni correction method. Statistical analyses were performed using SPSS Statistics (version 24.0; IBM Corp), and statistical significance was set at *P*<.05.

### Ethical Considerations

This study analyzed publicly available anti–substance use PSAs and did not involve human participants or the collection of identifiable personal data. Therefore, ethics approval was not required in accordance with institutional and international guidelines for research involving publicly accessible materials.

## Results

A total of 103 anti–substance use PSAs were initially identified. After screening, 14 PSAs were excluded because they were not in Chinese, were not produced by an organizational entity based in mainland China, or did not focus on anti–substance use. The final analytic sample consisted of 89 PSAs. The final sample included 15 antialcohol PSAs, 42 antidrug PSAs, and 32 antismoking PSAs. Together, these PSAs represented a diverse set of publicly available anti–substance use messages produced in mainland China and disseminated through multiple online and institutional channels.

Research Question 1 asked what the characteristics of anti–substance use PSAs in China were. In terms of the sponsoring organization of the PSAs, it was found that the antidrug units within the public security departments published the highest number of PSAs on anti–substance addiction (n=37, 41.2%), followed by the Chinese government (n=28, 31.5%), Chinese media (n=27, 26.9%), public welfare organizations (n=14, 15.7%), and commercial enterprises (n=8, 9%). It is also important to note that public health agencies did not issue any PSAs addressing anti–substance addiction. Among all sampled PSAs, the majority lacked a clearly defined target audience (n=54, 60.7%). Among those PSAs that did specify a target audience, the largest subset aimed at young and middle-aged individuals (n=30, 33.7%), followed by PSAs aimed at minors and older adults (n=3, 3.4%). Regarding the substance addictions highlighted in the PSAs (including smoking, drinking, and drug use), all PSAs focused solely on 1 single type of substance addiction (ie, smoking, drinking, or drug use). Regarding the framing strategies, 57% (n=6) of PSAs were negatively framed, while 48.3% (n=43) were positively framed. In terms of the presence of substance cues, 34.8% (n=31) of PSAs included substance cues. Regarding the cultural values, 60.7% (n=54) of PSAs emphasized the importance of eliminating substance use behaviors for the greater good of others and the whole country, reflecting a collectivist cultural perspective. In contrast, 48.3% (n=43) of PSAs focused on avoiding substance use behaviors for the individual’s own benefit, highlighting an individualistic cultural perspective. Only 9% (n=8) of PSAs incorporated both collectivist and individualistic cultural perspectives. Refer to Table 2 for more details.

**Table 2.** Frequencies of message design features and extended parallel process model (EPPM) and elaboration likelihood model (ELM) constructs in anti–substance use public service announcements from mainland China (N=89).

Variable	Values, n (%)
Sponsoring organization	
Public security departments	37 (41.6)
Chinese government	28 (31.5)
Chinese media	26 (26.9)
Public welfare organizations	14 (15.7)
Commercial enterprises	8 (9)
Public health agency	0 (0)
No sponsoring organization	6 (6.7)
Target audience	
Minors	12 (13.5)
Young and middle-aged individuals	30 (33.7)
Older adults	3 (3.4)
No clearly defined target audience	54 (60.7)
The presence of substance cue	
Absence	58 (65.2)
Presence	31 (34.8)
Addiction intervention scope	
Single substance addiction	89 (100)
Polysubstance addiction	0 (0)
Framing strategy	
Positive framed	64 (57)
Negative framed	43 (48.3)
Both	—^a^
Cultural value	
Collectivist	54 (60.7)
Individualistic	43 (48.3)
Both	8 (9)
EPPM constructs	
Threat appraisal	
Perceived Severity	55 (61.8)
Perceived Susceptibility	34 (38.2)
Both	21 (43.6)
Efficacy appraisal	
Response efficacy	63 (70.8)
Self-efficacy	45 (50.6)
Both	44 (49.4)
ELM constructs	
Central route cues	16 (18)
Peripheral cues	79 (88.8)
Both	6 (6.7)

a Not applicable.

Research Question 2 explored the extent to which anti–substance abuse PSAs in China conveyed threat appraisal (severity and susceptibility) and efficacy appraisal (self-efficacy and response efficacy). Our results indicated that regarding efficacy appraisal, 50.6% (n=45) of PSAs included self-efficacy information, while 70.8% (n=63) mentioned response efficacy. Almost half of PSAs (n=44, 49.4%) included information on both self-efficacy and response efficacy. Concerning threat appraisal, 61.8% (n=55) of PSAs provided information on perceived severity, while perceived susceptibility was mentioned in 38.2% (n=34) of PSAs. Both perceived severity and perceived susceptibility were included in 43.6% (n=21) of PSAs.

Research Question 3 examined the extent to which anti–substance abuse PSAs in China used central route cues and peripheral cues. Our results showed that the majority of sampled PSAs used peripheral route cues (n=79, 88.8%), followed by central route cues (n=16, 18%). In total, 6.7% (n=6) of PSAs used both peripheral route and central route cues.

Research Question 4 tested how the characteristics of anti–substance use PSAs, as well as the EPPM and ELM constructs, varied by different types of PSAs (including antialcohol, antitobacco, and antidrug PSAs). Holm–Bonferroni–adjusted P values are reported and interpreted in the following Results section; unadjusted or raw *P* values are provided in Table S2 in Multimedia Appendix 1. After applying the Holm-Bonferroni correction, our results indicated that PSA type was significantly associated with the sponsoring organization for public security departments and Chinese media, as well as with perceived susceptibility (all adjusted *P*=.01). As shown in Table 3, antidrug PSAs were predominantly produced by public security departments (37/42, 88.1%), whereas such sponsorship was not observed among antialcohol or antitobacco PSAs. In contrast, Chinese media sources were more frequently represented among antialcohol (8/15, 53.3%) and antitobacco PSAs (14/32, 43.8%) than among antidrug PSAs (2/42, 4.8%). In addition, perceived susceptibility cues were more prevalent in antismoking PSAs (20/32, 62.5%) than in antialcohol (6/15, 40%) and antidrug PSAs (8/42, 19%).

**Table 3.** Distribution of message design features and theoretical constructs across public service announcement (PSA) types, with Holm-Bonferroni adjusted *P* values.

Variables	Antialcohol PSAs, n (%)	Antitobacco PSAs, n (%)	Antidrug PSAs, n (%)	Holm-adjusted *P* value
Sponsoring organization				
Public security departments	0 (0)	0 (0)	37 (88.1)	.01
Chinese government	10 (66.7)	14 (43.8)	12 (28.6)	.28
Chinese media	8 (53.3)	14 (43.8)	2 (4.8)	.01
Commercial enterprises	4 (26.7)	3 (9.4)	1 (2.4)	.15
Framing strategy				
Positive framing	10 (66.7)	9 (28.1)	24 (57.1)	.12
Negative framing	11 (73.3)	24 (75)	22 (52.4)	.50
Substance cue (presence vs absence)	7 (46.7)	15 (46.9)	9 (21.4)	.28
Cultural value				
Collectivist cues	8 (53.3)	25 (78.1)	21 (50)	.28
Individualistic cues	9 (60)	13 (40.6)	21 (50)	.8
EPPM^a^ constructs				
Threat appraisal				
Perceived severity	12 (80)	17 (53.1)	26 (61.9)	.8
Perceived susceptibility	6 (40)	20 (62.5)	8 (19)	.01
Efficacy appraisal				
Response efficacy	12 (80)	19 (59.4)	32 (76.2)	.09
Self-efficacy	12 (80)	17 (53.1)	26 (61.9)	.09
ELM^b^ constructs				
Central route cues	2 (13.3)	11 (34.4)	3 (7.1)	.09
Peripheral route cues	14 (93.3)	26 (81.3)	39 (92.9)	.8

a EPPM: extended parallel process model.

b ELM: elaboration likelihood model.

Other associations, including those involving the Chinese government, commercial enterprises, framing strategies, substance cues, cultural values, self-efficacy, and ELM constructs, did not remain statistically significant after adjustment (all adjusted *P*>.05). However, descriptive patterns indicated that antialcohol (10/15, 66.7%) and antidrug (24/42, 57.1%) PSAs tended to use positive framing more frequently than antismoking PSAs (9/32, 28.1%) and that substance cues were less common in antidrug PSAs (9/42, 21.4%) than in antialcohol (7/15, 46.7%) and antitobacco PSAs (15/32, 46.9%). Antismoking PSAs also showed higher use of collectivist appeals (25/32, 78.1%) and central route cues (11/32, 34.4%) compared to the other PSA types.

## Discussion

### Principal Findings

This study examined the message design features and theory-based persuasive strategies used in anti–substance use PSAs in China. Overall, the findings reveal several key patterns in how these messages are constructed. First, PSAs showed limited audience targeting and were primarily developed by government-related organizations, with public health agencies notably absent. Messages tended to rely on negative framing and emphasized collectivist appeals. Second, the application of theory-based constructs was partial. PSAs relied heavily on peripheral cues, while central route cues were relatively limited. Similarly, EPPM components were not consistently applied, with greater emphasis on perceived severity and response efficacy than on susceptibility and self-efficacy. Finally, message design and persuasive strategies showed variation across different types of PSAs, although only a subset of these differences remained statistically significant after adjustment.

### Interpretation of Findings

The findings of this study highlight several broad patterns in the design of anti–substance use PSA in China. First, sponsorship was concentrated among government-related organizations, especially public security agencies, whereas public health agencies were absent. At the same time, most PSAs lacked a clearly defined target audience. Considered together, these patterns point to a structural gap in substance use prevention, where messaging appears to be driven more by administrative priorities than by established health communication principles. Despite audience segmentation being crucial for effective health campaigns [28], most PSAs lacked a clear target audience, with older adults being largely overlooked. According to data from the Chinese Center for Disease Control and Prevention, more than 36.5% of the older adult population (aged ≥60 years) consume alcohol [33], while more than 45.5% of older males in the same age group smoke [34]. By overlooking older adults, the Chinese government and public health professionals missed the chance to address substance use issues such as alcohol and tobacco, which have serious health risks. In addition, the greater use of negative framing, the limited depiction of substance use, and the predominance of collectivist appeals indicate that these PSAs tend to frame substance avoidance in terms of harm prevention and social responsibility rather than individualized choice.

The findings also indicated only partial use of theory-based message design. Consistent with prior research [20,22,24], the sampled PSAs relied much more heavily on peripheral cues than on central route cues, suggesting a preference for memorable, affective, or source-based appeals over evidence-based argumentation. A similar pattern was observed in the application of EPPM-based message design. Our findings indicated a partial application of EPPM-based message design, with only about half of the PSAs incorporating both threat and efficacy components, which is consistent with prior research [20]. Perceived severity and response efficacy appeared more frequently than perceived susceptibility and self-efficacy, suggesting that the messages more often communicated that substance use is harmful and that quitting is beneficial rather than emphasizing audiences’ personal risk and their ability to take action. Given that self-efficacy is critical for initiating and sustaining behavior change, this imbalance may limit the persuasive impact of these messages. Overall, these results suggest that although the ELM and EPPM are reflected in current PSAs, their application remains partial and uneven rather than systematic or theoretically integrated. However, it should be noted that only some theory-related associations (eg, perceived susceptibility) remained statistically significant after correction for multiple testing, whereas others (eg, self-efficacy and central route cues) did not. These results should be interpreted as exploratory patterns that require further validation.

Finally, differences across antialcohol, antitobacco, and antidrug PSAs suggest that anti–substance use communication in China does not adhere to a single persuasive logic but instead may vary by substance type. Antidrug PSAs were predominantly associated with public security sponsorship, whereas antialcohol and antitobacco PSAs were more frequently linked to Chinese media sources. In addition, several patterns were observed across PSA types. Antidrug PSAs appeared less likely to include substance depictions or self-efficacy cues, whereas antismoking PSAs showed higher use of collectivist appeals, perceived susceptibility, and central route cues. Antialcohol and antidrug PSAs also tended to use positive framing more frequently than antismoking PSAs. These patterns may reflect the varied social meanings, regulatory frameworks, and communicative roles associated with different substances in China. However, given the exploratory nature of these findings, further research is needed to validate these differences.

### Implications for PSA Design

First, the findings highlight the need to move beyond undifferentiated messaging toward more audience- and context-sensitive PSA design. The lack of clear audience targeting, together with the observed variation across PSA types, suggests that anti–substance use messages may be more effective when they are aligned with the characteristics of specific audiences and the risk profiles of specific substances. This is particularly important in substance use prevention, where different population groups may vary in their motivations for use, perceptions of risk, information-processing styles, and social contexts. More targeted PSA design may therefore enhance message relevance and improve the likelihood of effective communication.

Second, the findings suggest the importance of applying the EPPM and ELM in a more balanced and theoretically consistent manner. The current emphasis on perceived severity and response efficacy, together with the limited use of perceived susceptibility, self-efficacy, and central route cues, indicates that many PSAs may not fully engage the cognitive and motivational processes needed for persuasion and behavior change. Future PSA design may benefit from combining threat information with stronger efficacy support, while also incorporating clearer arguments, more concrete behavioral guidance, and higher-quality informational content.

Third, the findings suggest that current PSA strategies may be constrained by a single substance framework. Given the prevalence of polysubstance use in real-world contexts, where individuals often engage in overlapping or concurrent substance use, a narrow focus on one substance at a time may limit message relevance and reduce the ability of PSAs to address more complex patterns of use.

In addition, the prominence of collectivist appeals suggests that current PSAs may rely heavily on socially oriented framing. Although this approach may be appropriate in many cases, it may not be equally resonant across all audience segments. Future message design may therefore benefit from greater flexibility in balancing collectivist and individual-level appeals according to the target audience and communication context.

Finally, the dominance of government and public security agencies, together with the absence of public health sponsors, points to the importance of institutional diversity in PSA development. Broader participation by public health agencies and other prevention-oriented actors may support the development of messages that are not only administratively visible but also more evidence-based, behaviorally specific, and aligned with health communication principles.

### Limitations

Limitations of this study should also be noted. First, this study is among the first to examine the characteristics of anti–substance use PSAs and how these PSAs were developed using the ELM and EPPM as references. Given its exploratory nature, future studies are needed to assess the impact of these PSAs on actual attitude and behavior change, as our descriptive approach does not permit such claims. Second, the sample size is limited. However, this study included all anti–substance PSAs available for download, which are broadcast both online and offline nationwide. The limited sample size is largely due to the relatively few PSAs produced in China. In addition, the use of multiple statistical comparisons in this study increases the risk of type I error. Although *P* values were adjusted using the Holm-Bonferroni method to control for family-wise error rate, only a subset of associations remained statistically significant after correction. Therefore, some findings, particularly those initially identified at the conventional *P*<.05 level, should be interpreted with caution and considered exploratory. These patterns may need further investigation and replication in future studies.

### Conclusions

This exploratory study provides a theory-informed analysis of anti–substance use PSAs in China by examining both message design features and the application of constructs from the ELM and EPPM. The findings suggest that current PSAs rely on only partial and uneven use of theory-based persuasive strategies, while also showing limited audience targeting and certain variation across substance types. Taken together, the findings of this study highlight 3 key patterns in the design of anti–substance use PSAs in China: limited audience segmentation, partial and uneven application of theory-based persuasive strategies, and substance-specific differences in message design and persuasion strategies. These patterns suggest that current PSA design may be constrained not only by incomplete application of theoretical frameworks but also by institutional structures, cultural orientations, and the varied social positioning of specific substances. These insights point to the need for PSA design approaches that are context sensitive and theory informed, taking into account audience diversity, institutional dynamics, and the specific social and regulatory contexts of different substances, including polysubstance use, which is often overlooked.

## Supplementary material

10.2196/85703 Multimedia Appendix 1 Detailed search strategy and supplementary statistical analyses for anti–substance use public service announcements identified in mainland China.

## Abbreviations

ELM: elaboration likelihood model
EPPM: extended parallel process model
PSA: public service announcement

## References

[1] (2025). Tobacco. World Health Organization.

[2] (2024). Global status report on alcohol and health and treatment of substance use disorders. World Health Organization.

[3] Li XH (2020). 2018 China Adult Tobacco Survey Report.

[4] (2018). Global status report on alcohol and health 2018. World Health Organization.

[5] (2024). 2023 China drug situation report. http://www.nncc626.com/20240619/d1a1ffb1f3fb4c93bb05f6e73fb50414/c.html.

[6] Allara E, Ferri M, Bo A, Gasparrini A, Faggiano F (2015). Are mass-media campaigns effective in preventing drug use? A Cochrane systematic review and meta-analysis. BMJ Open.

[7] Durkin S, Brennan E, Wakefield M (2012). Mass media campaigns to promote smoking cessation among adults: an integrative review. Tob Control.

[8] Tay R (2005). Mass media campaigns reduce the incidence of drinking and driving. Evid Based Healthc Public Health.

[9] Puppin G (2020). Forty years of the return of advertising in China (1979–2019): a critical overview. JOMEC J.

[10] Yang G (2018). Tobacco Control in China.

[11] Petty RE, Cacioppo JT, Cacioppo JT, Petty RE (1986). Communication and Persuasion.

[12] Popova L (2012). The extended parallel process model: illuminating the gaps in research. Health Educ Behav.

[13] Witte K (1994). Fear control and danger control: a test of the extended parallel process model (EPPM). Commun Monogr.

[14] Chen L, Yang X, Fu L, Liu X, Yuan C (2019). Using the extended parallel process model to examine the nature and impact of breast cancer prevention information on mobile-based social media: content analysis. JMIR Mhealth Uhealth.

[15] Kim H, Lee D, Hong Y, Ahn J, Lee KY (2016). A content analysis of television food advertising to children: comparing low and general‐nutrition food. Int J Consum Stud.

[16] Scannell D, Desens L, Guadagno M (2021). COVID-19 vaccine discourse on Twitter: a content analysis of persuasion techniques, sentiment and mis/disinformation. J Health Commun.

[17] Shi JJ, Wang XV, Peng TQ, Chen L (2019). Cancer-prevention messages on Chinese social media: a content analysis grounded in the extended parallel process model and attribution theory. Int J Commun.

[18] Zou W, Zhang WJ, Tang L (2021). What do social media influencers say about health? A theory-driven content analysis of top ten health influencers’ posts on Sina Weibo. J Health Commun.

[19] Lam C, Huang Z, Shen L (2022). Infographics and the Elaboration Likelihood Model (ELM): differences between visual and textual health messages. J Health Commun.

[20] Niederdeppe J, Avery RJ, Miller EE (2018). Theoretical foundations of appeals used in alcohol-abuse and drunk-driving public service announcements in the United States, 1995-2010. Am J Health Promot.

[21] Turner M, Boudewyns V, Kirby-Straker R, Telfer JL (2013). A double dose of fear: a theory-based content analysis of news articles surrounding the 2006 cough syrup contamination crisis in Panama. Risk Manag.

[22] Ledford CJ (2009). Content analysis of internet marketing strategies: how pharmaceutical companies communicate about contraceptives with consumers online. Soc Mark Q.

[23] Manganello JA, Clegg Smith K, Sudakow K, Summers AC (2013). A content analysis of food advertisements appearing in parenting magazines. Public Health Nutr.

[24] Morimoto M (2017). Examining information contents and ad appeals in Japanese over-the-counter drug advertising. Int J Commun Health.

[25] Van Deventer D, Marecaux J, Doubleday A, Errett N, Isaksen TM (2021). Wildfire smoke risk communication efficacy: a content analysis of Washington State's 2018 statewide smoke event public health messaging. J Public Health Manag Pract.

[26] Goldsmith RE, Lafferty BA, Newell SJ (2000). The impact of corporate credibility and celebrity credibility on consumer reaction to advertisements and brands. J Advert.

[27] Goldsmith R, Lafferty B, Newell S (2000). The influence of corporate credibility on consumer attitudes and purchase intent. Corp Reput Rev.

[28] Slater MD (1996). Theory and method in health audience segmentation. J Health Commun.

[29] Liu J, Bailey RL (2019). Effects of substance cues in negative public service announcements on cognitive processing. Health Commun.

[30] Wong NC, Harrison KJ, Harvell LA (2015). Reactance and public health messages: the unintended dangers of anti-tobacco PSAs. Stud Media Commun.

[31] Xiao R, Li C (2022). The influence of China’s practice of collectivism on its COVID-19 policies and public attitude. J Sociol Ethnol.

[32] Landis JR, Koch GG (1977). The measurement of observer agreement for categorical data. Biometrics.

[33] Li YR, Wang J, Zhao LY (2018). The drinking status and associated factors in adults in China [Article in Chinese]. Zhonghua Liu Xing Bing Xue Za Zhi.

[34] (2021). China Chronic Disease and Risk Factor Surveillance Report 2018.

