# Supplementary material for: Decoding Anti–Substance Use Public Service Announcements: Content Analysis Grounded in the Elaboration Likelihood Model and Extended Parallel Process Model
Source: JMIR Form Res. 2026 May 14;10:e85703. doi: 10.2196/85703 (PMC13175447; doi:10.2196/85703)
Supplement: Multimedia Appendix 1 [file formative-v10-e85703-s001.docx]

Table S1: Detailed Search Strategy for Identification of Anti-Substance Use Public Service Announcements in Mainland China

| **Platform** | **Search Dates** | **Search Term** | **Example Queries** |
| --- | --- | --- | --- |
| Bilibili | 12/21/2023 – 01/11/2024 | drinking (饮酒), alcohol consumption (喝酒), alcohol (酒/酒精); smoking (吸烟/抽烟), tobacco (烟/烟草); drugs/anti-drug (毒品/禁毒); public service announcement (公益广告/公益宣传片) | anti-drug PSA (禁毒 公益广告); anti-smoking PSA (吸烟 公益广告); alcohol-related PSA (酒精 公益宣传片) |
| iQIYI | 12/21/2023 – 01/11/2024 | Same as above | Same as above |
| Youku | 12/21/2023 – 01/11/2024 | Same as above | Same as above |
| Tencent Video | 12/21/2023 – 01/11/2024 | Same as above | Same as above |
| Chinese media websites (e.g., official websites of national and provincial television networks and news organizations) | 02/09/2024 – 02/26/2024 | Same as above | Same as above |
| Government and institutional websites (e.g., anti-drug offices, public security agencies, CDC, etc.) | 03/05/2023 – 04/21/2024 | Same as above | Same as above |

| Table S2: Holm-Bonferroni adjusted *p*-values for associations between PSA type and message design features, EPPM and ELM constructs. | | | |
| --- | --- | --- | --- |
| Variables | *X*^2^ (df = 2) | Raw  *p*-value | Holm-adjusted  *p*-value |
| Sponsoring organization |  |  |  |
| Public security departments | 82.347 | < .001 | .015 |
| Chinese government | 6.472 | .037 | .288 |
| Chinese media | 22.204 | < .001 | .015 |
| Commercial enterprises | 6.938 | .017 | .153 |
| Framing strategy |  |  |  |
| Positive framing | 8.514 | .012 | .12 |
| Negative framing | 4.531 | .1 | .5 |
| Substance cue  (presence vs. absence) | 6.398 | .044 | .288 |
| Cultural value |  |  |  |
| Collectivist cues | 6.54 | .036 | .288 |
| Individualistic cues | 1.619 | .455 | .8 |
| EPPM constructs |  |  |  |
| Threat appraisal |  |  |  |
| Perceived severity | 3.053 | .205 | .8 |
| Perceived susceptibility | 14.598 | <.001 | .015 |
| Efficacy appraisal |  |  |  |
| Response efficacy | 3.026 | .2 | .8 |
| Self-efficacy | 9.808 | .008 | .096 |
| ELM constructs |  |  |  |
| Central route cues | 8.798 | .009 | .099 |
| Peripheral route cues | 2.48 | 0.26 | .8 |
